# Moisture-enabled electricity generation

**DOI:** 10.1093/nsr/nwaf171

**Published:** 2025-04-25

**Authors:** Puying Li, Huhu Cheng, Zhenzhong Yang, Liangti Qu

**Affiliations:** State Key Laboratory of Flexible Electronics Technology, Key Laboratory of Organic Optoelectronics & Molecular Engineering, Ministry of Education, Department of Chemistry, Tsinghua University, China; State Key Laboratory of Flexible Electronics Technology, Key Laboratory of Organic Optoelectronics & Molecular Engineering, Ministry of Education, Department of Chemistry, Tsinghua University, China; Department of Chemical Engineering, Tsinghua University, China; State Key Laboratory of Flexible Electronics Technology, Key Laboratory of Organic Optoelectronics & Molecular Engineering, Ministry of Education, Department of Chemistry, Tsinghua University, China

## Abstract

This perspective underscores the core challenges and primary requirements of moisture-enabled electric generators, and further provides guidelines for future research.

The atmosphere stores a large amount of water vapor, reaching 12 900 km^3^. The phase conversion process of water transitioning from the gaseous state to the adsorbed state entails significant energy changes, according to a Gibbs energy variation of 8 kJ mol_water_^−1^, an enthalpy variation of 40.66 kJ mol_water_^−1^, and an entropy variation of 109.0 J mol_water_^−1^ K^−1^. This energy was not effectively utilized until the proposal of moisture-enabled electric generators (MEGs) in 2015 [1]. The electricity generation of MEGs is related to the directed diffusion of charged ions in functional material after moisture absorption. The ubiquitous energy and the simple energy harnessing mode have garnered a lot of attention from researchers, and offer potential for building large-scale MEG arrays to fulfill the goal of an off-grid and portable power supply. Thanks to years of efforts, most MEGs can generate considerable electricity under a relative humidity ranging from 20%∼95% RH and a temperature between 0–60°C, nearly achieving round-the-clock and all-region-applicable electricity generation. The power density of macro-scale MEGs has been improved to ∼0.1 W m^−2^, while fiber-based MEGs achieve up to 1 W m^−2^, and quantum-dot-based micro-MEGs reach as high as ∼20 W m^−2^. The electricity generation period in a single moisture absorption cycle has been lengthened from seconds to hundreds of hours [2]. Despite the great progress made so far, the energy conversion efficiency of the currently available MEGs is relatively low (generally less than

10%), and there exists a performance gap between macro- and micro-scale MEGs, calling for the innovation of MEG technology from device unit to integrated system. From this perspective, we underscore the core challenges and primary requirements of MEGs and provide guidelines for future research (Fig. 1).

**Figure 1.**
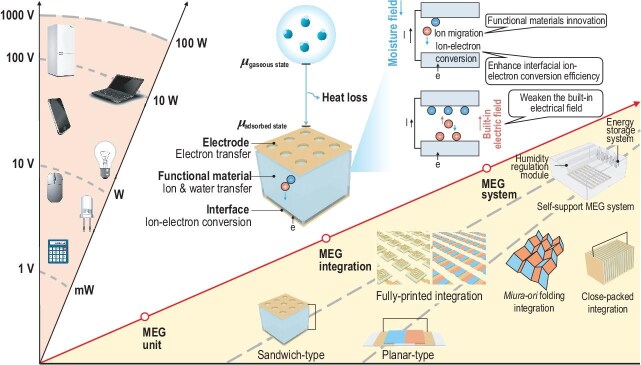
A schematic diagram of the electricity generation process of MEGs and the key stages in the progression of MEGs towards practical application, from the MEG unit and MEG integration to the MEG system. *μ* is the chemical potential.

Power density stands as one of the pivotal indicators for green energy harvesters. Contemporary MEGs have achieved direct drive for small-scale electrical appliances such as LEDs and table lamps. However, for common household appliances, the power density needed is 50∼100 W. Such a gap between energy demand and supply makes MEG innovation an imperative.

Functional material innovation represents a widely adopted solution. One of the key approaches is to regulate the chemical composition of functional materials. Enhancing the material's moisture-absorption ability can increase the amount of water molecules involved in the phase transition, thereby increasing the input energy [3]. Regulating the functional groups promotes ionic dissociation for enhanced quantity and charge density of migrated ions. Ions featuring a small ionic radius and low ionic valence exhibit low electrostatic repulsive force to attain efficient ion diffusion, while high-valent ions may possess high charge density. Another key approach is to construct a chemical and physical structure to facilitate ion migration. A chemically asymmetric structure can be constructed, for instance, by pre-introducing a functional group gradient [4] or constructing a Janus charge heterostructure [2]. This facilitates the establishment of an ion concentration gradient and further enhances the driving force for ion migration. The fabrication of channels for the rapid transport of water and ions at the level of molecular chain, nanometer and micrometer scales may also be considered [5]. However, the ion migration within functional materials subsequent to moisture absorption merely constitutes one step in the electricity generation process. This implies that efforts only focusing on functional materials innovation cannot reach the upper limit of energy conversion. Meanwhile, the approach grounded in functional material innovation usually leads to a trade-off between the power density and electricity generation time of MEGs. For example, reducing the ion migration resistance of the functional material invariably accelerates the equilibrium time of ionic thermal motion which leads to faster electric signal decay.

In contrast, ion-electron conversion at the functional material/electrode interface has received scant attention. The ion-electron conversion not only serves as the direct process of electricity generation, but also influences the built-in electric field, which determines the attenuation of electric signal. Therefore, through enhancing the interfacial ion-electron conversion, both the power density and the electricity generation time can be theoretically enhanced. It is worth noting that the introduction of active reactive electrodes into MEGs to boost the electricity generation should be avoided, because the irreversible chemical corrosion reaction is contrary to the original intention underlying the development of green energy. It is a rational approach to decorate high-capacity materials on electrodes to increase the ion storage sites. The augmentation of ion-electron interaction sites facilitates the interfacial ion-electron conversion. This, in turn, alleviates electrode polarization, thereby inhibiting the formation of the built-in electric field and prolonging the electricity generation period.

Minimizing energy losses during the electricity generation process also represents a crucial approach to improving the power density. Adding hydrophilic groups and increasing surface roughness on the functional materials can weaken the intermolecular forces that water molecules must overcome to enter the functional material. Reducing the swelling ratio of the functional materials can reduce the energy dissipation caused by volume expansion after water absorption.

The slow electricity-generation-performance decline after repeated water absorption and dehydration has long been neglected. This may be attributed to the large ionic resistance of functional material. Some ions are unable to return to the initial position after dehydration. This leads to a reduction in the number of ions participating in ion migration within the MEGs upon moisture re-absorption. In addition, the structural failure of functional material, which results in a decrease in water-absorption capacity and an increase in ionic resistance, also contributes to the performance decline. Therefore, the preparation of functional materials featuring low ionic resistance, stable structure and components is crucial for resolving this issue.

Apart from the improvement of the single MEG unit, MEG unit integration is essential for practical applications. The development of device configurations that are conducive to efficient, compact and fully printed integration is needed. In the early stages, MEGs adopted a sandwich-type device configuration for the construction of a moisture field [1]. Although the small-scale series and parallel integration has been achieved, when the sandwich structure evolves towards full-printed integration, MEGs may encounter the issues of electrode cracking and poor inter-unit connection during electricity generation due to the mechanical property differences between the electrode and the functional material [6]. Later, planar-type MEGs were developed. Compared to the sandwich-type device configuration, the planar-type MEGs are more suitable for full printing [7]. Hundreds of volts of voltage and milliamperes of current can be easily realized through rapid printed integration. However, directional ion migration between the electrode pair is difficult to construct in planar-type MEGs. Therefore, asymmetric chemical and physical structures need to be constructed in advance [7]. Meanwhile, a larger distance between the electrode pair in planar-type MEGs directly causes a larger ionic resistance in the MEGs unit. Furthermore, the planar device structure suffers from suboptimal space utilization. It is of great necessity to develop MEGs that can fully utilize the moisture energy and achieve a high integration level, like *Miura-ori* folding or close-packed integration.

In addition, the development of MEGs customized to specific application scenarios is insufficient. First, there are a variety of climates and geographical environments around the world, such as low humidity and large temperature differences, while the majority of research on MEGs has been conducted in high-humidity and room-temperature conditions. Therefore, developing materials with tailored properties customized to specific environments is of critical importance, for instance, for ultra-low humidity climates, developing functional materials with a high water-absorption efficiency and capacity, or increasing the quantity of ions to counteract the performance degradation resulting from the high internal resistance of ion migration [2]. To adapt to ultra-low-temperature environments, low-freezing-point components can be incorporated into the functional materials. Second, flexible wearables are in high demand due to the explosion of the Internet of Things, while high-performance flexible MEGs have not been developed. Apart from the trade-offs between the electrochemical and mechanical performance of materials that flexible devices typically encounter, the equilibrium between the water/gas permeability of wearables and the hydroscopicity necessary for MEGs’ electricity generation needs to be considered. The development of materials and structures that retain good permeability after water absorption is essential. Taking into account the potential health hazards associated with skin contact with MEGs, it is imperative to select functional materials with low biotoxicity, or utilize biocompatible packaging materials such as fibroin protein coatings to isolate potential hazards. Moreover, for prospective applications in areas like deep-sea research and intelligent healthcare, it is necessary to develop MEG functional materials that possess high-pressure resistance and biocompatibility.

Furthermore, due to the environmental humidity fluctuations and the high-temperature or low-humidity environment required for MEG regeneration, achieving stable power output without human interference necessitates the development of the MEG system. MEGs could be integrated with a humidity self-regulating module to achieve autonomous moisture absorption and desorption. An energy storage system may also be coupled with an MEG to mitigate the variations in electric signals induced by environment condition fluctuations [8]. In addition, MEGs can also be combined with other green energies to address the issue of electric signal fluctuations caused by environmental variations [9].

The development of MEG technology needs interdisciplinary collective efforts in different fields, including materials science, chemistry, physics, manufacturing and electrical engineering. There are also broad prospects for the combination of emerging artificial intelligence and MEGs. For example, machine-learning models can be employed to decouple the mixed signals from synchronous multimode monitoring, thereby facilitating the construction of self-powered multimode sensors based on MEGs [10]. Machine learning can also analyze the existing experimental data, to predict stable and efficient materials and structures for MEGs. Reinforcement learning can be integrated with meteorological data to optimize the operating hours and replacement intervals of MEGs. Meanwhile, green energy development necessitates that the entire lifecycle of MEGs, from usage to recycling, adhere to environmental footprint standards based on sustainable materials. The synergy between industrial R&D and academic research guarantees the market-demand-oriented development of MEGs. By virtue of these endeavors, we are convinced that MEGs can genuinely progress towards practical applications.
